# Cytotoxic Edema and Intra-parenchymal Hemorrhage: A Mediated Pathway to Mortality and Functional Outcome in Cerebral Venous Sinus Thrombosis- A sub-analysis of the CLOT-VENUS Registry

**DOI:** 10.1007/s12975-026-01426-9

**Published:** 2026-04-14

**Authors:** Nashwa Abdelhakim, Milagros Galecio-Castillo, Piyush Kalakoti, Leonardo Cruz-Criollo, Aaron Rodriguez-Calienes, Anderson Brito, Jorge Cespedes, Amir Shaban, Anish Venkatesan, Vanessa Cano Nigenda, Andres Alberto Mercado Pompa, Nicholas M. Mohr, Adrian Pereda-Castillo, Kevin Enríquez Peregrino, Hector Valdez Ruvalcaba, Brian J. Smith, James C. Torner, Miguel A. Barboza, Antonio Arauz, Santiago Ortega-Gutierrez

**Affiliations:** 1 Department of Neurology, University of Iowa Health Care, Iowa City, IA USA; 2 Universidad Científica del Sur, Lima, Perú; 3 Instituto Nacional de Neurologia y Neurocirugía Manuel Velasco Suárez, México City, México; 4 Departments of Emergency Medicine, University of Iowa Carver College of Medicine, Iowa City, IA USA; 5 Department of Epidemiology, College of Public Health, University of Iowa, Iowa City, IA USA; 6 Division of Critical Care, Department of Anesthesia, University of Iowa Carver College of Medicine, Iowa City, IA USA; 7 Department of Biostatistics, University of Iowa, Iowa City, IA USA; 8 Department of Neuroscience, Hospital Dr. Rafael A. Calderon Guardia, San Jose, Costa Rica; 9 Department of Neurosurgery, University of Iowa Health Care, Iowa City, IA USA; 10 Department of Radiology, University of Iowa Health Care, Iowa City, IA USA

**Keywords:** Cerebral venous thrombosis, Cytotoxic edema, CVT, Intraparenchymal Hemorrhage

## Abstract

Cytotoxic edema (CE) is a radiographic marker of early tissue injury in cerebral venous thrombosis (CVT) associated with poor outcomes, yet its mechanistic pathway remains unclear. We investigated whether intraparenchymal hemorrhage (IPH) mediates the association between CE and outcomes. We conducted a retrospective cohort study using the multicenter CLOT-VENUS registry, including acute CVT patients treated at two Comprehensive Stroke Centers in the USA and Mexico (2004–2024). CE was defined as hyperintensities around IPH or venous infarct with DWI restricted diffusion and corresponding low ADC, confirming true restricted diffusion. IPH was defined as hemorrhagic transformation of venous infarction or intracerebral hemorrhage on GRE MRI and/or NCCT. Mediation analyses assessed whether IPH mediated CE associations with in-hospital mortality and functional outcomes. Among 394 patients (mean age 42.7 years; 65.5% female), 128 (32.5%) demonstrated CE and 111(30.2%) IPH. CE was associated with in-hospital mortality (aOR 2.63, 95% CI 1.01–7.12) and poor 6-month mRS (aOR 1.71, 95% CI 1.06–2.74). CE was associated with IPH, which in turn was associated with mortality (aOR 8.73, 95% CI 2.93–30.45) and poor mRS (aOR 1.96, 95% CI 1.18–3.25). Adjustment for IPH rendered the CE-outcome associations non-significant. IPH accounted for 76.8% of CE’s effect on mortality and 83.8% on 6-month mRS. Our findings suggest that IPH likely mediates the effect of CE on outcomes in CVT. Although the temporal sequence could not be confirmed, the results underscore the value of early CE detection for timely intervention.

**Supplementary Information:**

The online version contains supplementary material available at 10.1007/s12975-026-01426-9.

## Introduction

Cerebral venous thrombosis (CVT) is an uncommon but significant cause of stroke, accounting for 0.5–1% of all cerebrovascular events. It predominantly affects younger individuals, leading to morbidity and loss of productive life [1]. Cerebral venous infarction (CVI) occurs in nearly half of all CVT cases, [2] Intraparenchymal hemorrhage (IPH) is observed in up to 40% of patients [3, 4]. The presence of IPH at presentation is often associated with more severe clinical manifestations [5–7] and an increased risk of unfavorable outcomes [8]. Although long-term outcomes are often favorable, most deaths in CVT occur during the initial hospitalization despite anticoagulation therapy [9].

Various subtypes of edema can be observed in CVT, including: focal cytotoxic edema (CE), focal vasogenic edema (VE), and global cerebral edema (GCE) [10]. Experimental animal studies have demonstrated that CE is an early pathological event in the course of CVT, preceding the development of VE and hemorrhagic transformation [11, 12]. CE appears early in the disease course, characterized by reduced apparent diffusion coefficient (ADC), indicative of restricted diffusion due to neuronal injury and intracellular swelling [13]. In contrast, VE is typically secondary to increased venous pressure and blood-brain barrier (BBB) disruption, and emerges later [11, 12]. Despite these pathophysiological insights and evidence linking CE to poor prognosis in other cerebrovascular diseases, its role in CVT remains insufficiently understood [14–16]. In particular, its potential progression to IPH and association with clinical deterioration, probably as a result of localized ischemia and damage to intracellular ion channels, eventually leading to neuronal swelling, is a characteristic of cerebral venous involvement [15, 16]. Converging evidence from clinical studies, imaging, and animal models supports that CE exacerbates brain injury and worsens outcomes in CVT [13, 17–19]. A recent bibliometric analysis of landmark CVT research highlighted the limited investigation of CE and called for further research into this aspect of CVT pathophysiology [20].

We aimed to examine the relationship between CE, IPH, and outcomes in CVT patients, integrating advanced neuroimaging biomarkers and robust statistical approaches to provide novel insights into CVT pathophysiology and prediction.

## Methods

### Study Design and Data Source

This was a retrospective observational study using data from the “CoLlabOraTion on Cerebral VEnoUs ThrombosiS” CLOT-VENUS registry, which included 394 consecutive CVT patients treated between 2004 and 2024 at two Comprehensive Stroke Centers in the United States and Mexico. For this study, we included adult patients (*≥* 18 years) who were hospitalized with the diagnosis of acute non-iatrogenic or traumatic CVT with available acute neuroimaging (MRI and/or CT). Patients who had an initial encounter in an outpatient setting, were not hospitalized, or who did not have available acute neuroimaging studies were excluded. The patient selection algorithm is detailed in Supplementary Fig. 1.

The CLOT-VENUS registry was approved under the waiver of informed consent by the local Institutional Review Board (IRB) at the University of Iowa Health Care (IRB number: 202210065) and Instituto Nacional de Neurologia y Neurocirugia Manuel Velasco Suárez (IRB number: 25/23). The study adheres to the Strengthening the Reporting of Observational Studies in Epidemiology guidelines [21] and the recommendations outlined in A Guideline for Reporting Mediation Analyses (AGReMA) Statement [22]. The data supporting our results are available upon reasonable request to the corresponding author.

### Clinical and Radiological Variables

The CLOT-VENUS registry is described in detail in Supplementary Table 1. The dataset includes: (1) patient demographics: age, sex, race, body mass index (BMI); (2) predisposing risk factors: pregnancy or postpartum period, exogenous hormone exposure (oral contraceptive use [OCP]/hormone replacement therapy [HRT] was defined as active use at admission or within 30 days; when available, OCPs (combined vs. progestin-only) and HRT (oral vs. transdermal) were subtyped, smoking status, prior infections, stratified by site (central nervous system, ears/nose/throat, gastrointestinal, musculoskeletal, obstetric/gynecologic, respiratory, or urinary tract), dehydration, trauma (defined as a history of head or neck trauma prior admission), history of malignancy, systemic disorders (prior history of inflammatory bowel disease, Behcet Disease, Thyroid disease, Systemic Lupus, Antiphospholipid syndrome, Nephrotic syndrome, Sarcoidosis, and Granulomatosis with polyangiitis), and history of any neurosurgical intervention four weeks prior admission. (3) presenting symptoms at admission included headache, seizure, dysarthria, cranial nerve deficit, altered mental status (defined as disorientation on person, time, place, and/or incapability of follow commands, and based on the first item of the National Institutes of Health Stroke Scale [NIHSS] or the verbal/motor response item on the Glasgow Coma Scale [GCS]), [23, 24] motor and sensory deficits, abnormal coordination, abnormal reflexes, and Babinski sign; (4) neurological examination was quantified at baseline and follow-ups using the modified Rankin Scale (mRS) score [25] GCS scores were recorded at admission and stratified as follows: mild (GCS 13–15), moderate (9–12), and severe (GCS < 9) [24]. (5) In-hospital variables included laboratory values, treatment approaches such as anticoagulation regimens, and endovascular therapy (EVT) or decompressive craniectomy during hospitalization. Initial CVT severity was represented by clinical features at presentation, including altered mental status, seizure, and focal neurological deficits, features known to reflect disease severity and neurological impairment [26].

Neuroimaging includes brain magnetic resonance imaging (MRI) and/or non-contrast CT, as well as vascular images, MR or CT venograms. All admission neuroimaging studies performed within 24 h of hospital presentation were included in the imaging analysis. All scans were re-read de novo specifically for the CLOT-VENUS analysis. Images were independently reviewed in duplicate by two readers selected from a pool comprising: a neurovascular postdoctoral fellow with formal neuroimaging training (ARC), a board-certified vascular neurologist (AS), and a senior board-certified neurointerventionalist (SOG). To ensure duplicate reads for the entire cohort, SOG reviewed all cases, paired with AS for Iowa cases through October 2018 and with ARC for Iowa cases from November 2018 to 2024, and for all Mexico cases. All readers were blinded to clinical data and outcomes. Discrepancies were resolved by consensus, with the senior neurointerventionalist serving as adjudicator when needed.

Edema subtypes were defined a priori using established criteria: (a) *Focal Cytotoxic Edema (CE)* develops as intracellular swelling resulting from energy failure due to impaired venous outflow and subsequent hypoxia. It indicates early neuronal injury and may precede venous infarction and subsequent hemorrhagic transformation [12, 13]. It can also be found in the surrounding IPH regions independent of venous infarction. It was identified as hyperintensities indicating restricted diffusion on diffusion-weighted imaging (DWI) with corresponding hypointensities in the apparent diffusion coefficient (ADC), confirming true restricted diffusion [27]. In non-enhanced CT, it was characterized as focal edema in the venous vascular territory with subtle loss of gray-white matter interface (differentiation) due to the rise of water content [28, 29] (b) *Focal Vasogenic Edema (VE)* represents extracellular accumulation of fluid due to blood–brain barrier disruption. It was identified as hyperintensities on FLAIR MR sequences with normal or increased ADC values, localized around the venous clot visualized on gradient echo (GRE) or susceptibility-weighted imaging (SWI) [27]. It was also identified in non-contrast CT as hypoattenuation predominantly involving white matter with relative sparing of cortical gray matter [30] (c) *Global Cerebral Edema (GCE)* was defined as bilateral finger-like extensions into the gray-white junction with effacement of gyri and sulci on admission CT [31]. IPH was defined as visible hyperdensity on CT or hypointensity on GRE or SWI sequences [32] while venous infarct was identified by confluent parenchymal lesions by hyperintensity on DWI, hypointensity, or isointensity on the ADC map, and FLAIR/T2 hyperintensity [33, 34]. Subarachnoid hemorrhage (SAH) was defined as abnormal hyperdensity in the subarachnoid space on CT or hyperintensity in the subarachnoid space on FLAIR or hypointensity on GRE or SWI sequences [32]. Readers also recorded clot anatomical distribution, classified as cortical veins, dural sinuses (e.g., superior sagittal sinus, inferior sagittal sinus, transverse/sigmoid sinuses, and straight sinus), and deep cerebral venous system (e.g., internal cerebral veins, vein of Galen, and basal vein of Rosenthal) [35, 36]. We calculated an inter-rater reliability (IRR) coefficient using pairwise Cohen’s Kappa *(κ)*, [37] between the senior neurointerventionalist and the vascular neurologist were almost excellent for venous infarct (κ = 1.0), and IPH (κ = 1.0), and substantial for GCE (κ = 0.79), CE (κ = 0.76), and VE (κ = 0.65). IRR between the neurovascular research postdoctoral fellow and the neurointerventionalist were almost excellent for venous infarct (κ = 1.0) and IPH (κ = 1.0), and substantial for cytotoxic edema (κ = 0.82), global cerebral edema (κ = 0.69), and vasogenic edema (κ = 0.61). All p-values were < 0.05. We used a standard naming for level of reliability indicated by κ values: slight: <0.20, fair: >0.21–0.40, moderate: >0.41–0.60, substantial: >0.61–0.80, and almost perfect: >0.81-1.00.

### Outcome Measures

*The primary outcomes* included in-hospital mortality and functional outcome measured by the 6-month modified Rankin Scale (mRS) score ordinal shift distribution. *Secondary outcomes* included discharge ordinal mRS. Given that CVT cohorts are typically younger and less severely disabled than acute ischemic stroke (AIS), with a left-shifted mRS distribution, we prespecified two dichotomies: (1) functional independence (mRS 0–2 vs. 3–6), which is commonly used in the CVT literature for comparability; and (2) less-than-excellent vs. excellent outcomes (mRS 2–6 vs. 0–1), to better capture patients with near-baseline function in this population [25].

The mRS at discharge was derived from standardized evaluations in physical and occupational therapy performed prior to discharge. Follow-up mRS post-discharge was assessed during outpatient visits by certified clinical providers [38]. Due to heterogeneity in follow-up mRS assessments’ timepoints, the mRS statuses at 6 months were imputed from the observed visits [39, 40]. The mRS evaluations and imputation analysis are described in Supplementary Table 2.

### Mediation Analysis Framework

The mediation models assessed whether IPH acts as a mediator of the effect of CE (exposure) on three prespecified outcomes: in-hospital mortality, functional status at discharge, and functional status at 6 months. The assumed relationship (CE → IPH → outcome) can be conceptualized as a pathophysiological model, supported by previous evidence indicating that CE develops acutely after ischemia, while secondary IPH occurs later through BBB disruption [11–13, 19].

**Fig. 1:**
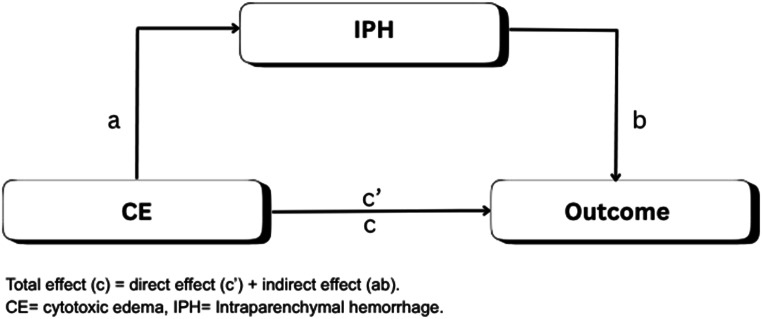
Model of the Hypothetical Mediation pathway in patients with CE

The mediation structure followed the framework outlined by Baron and Kenny, and by VanderWeele and Vansteelandt for binary (mortality) and ordinal (mRS) outcomes [41, 42]. Four pathways were tested (Fig. 1):

- **Pathway C**: CE → outcomes.
- **Pathway A**: CE → IPH.
- **Pathway B**: IPH → outcomes (adjusted for CE).
- **Pathway C′**: CE and IPH → outcome (simultaneously in the model).

A mediation effect was confirmed when the inclusion of IPH attenuated or eliminated the effect of CE on the outcomes. All pathways were tested using multivariable logistic or ordinal (proportional odds) regression analysis and reported as adjusted odds ratios (aORs) [41, 43].

### Statistical Analysis

Descriptive statistics were used to summarize continuous and categorical variables. For categorical variables, counts and percentages were reported, while mean (SD) or median (interquartile range) was used for continuous variables. Group comparisons between CVT patients with and without CE were performed using Pearson’s chi-square or Fisher test for categorical variables, depending on expected cell counts, and independent t-tests for continuous variables, assuming approximate normality under the Central Limit Theorem due to the large sample size.

To evaluate the association between CE and outcomes, several multivariable models were constructed. First, “candidate” variables were selected a priori based on their clinical relevance: Age, female sex, center, history of malignancy, history of systemic disorder, GCS, pre-existing infection, motor weakness, initial anticoagulation, seizure, clot location, and venous infarction. These variables were considered potentially informative at the time of admission. Although endovascular therapy and decompressive craniectomy could be viewed as potential confounders, both are indication-driven interventions initiated after baseline assessment. Accordingly, they were not included as candidate adjustment variables, as controlling for downstream treatments would condition on post-exposure factors and bias estimation of the total association between admission edema and outcome. Then, bidirectional Stepwise procedures were applied, and model selection was guided by the Akaike Information Criterion (AIC) for most outcomes, or the Bayesian Information Criterion (BIC)for rare events outcomes, to optimize model fit. This approach allowed for clinically meaningful yet statistically stable models. Logistic and ordinal (proportional odds) regression models were used to evaluate the association between CE and functional outcomes.

Following VanderWeele et al.‘s causal inference theory, the total effect (TE)represents the overall association between the exposure and the outcome. This effect can be decomposed into two components: the natural direct effect (NDE), which reflects the portion of the effect that is not mediated and operates independently of the mediator, and the natural indirect effect (NIE), which quantifies the portion of the effect caused by the mediator [44]. The NIE was calculated on the log-odds scale using the formula log (OR_NIE_) = log (OR_TE_) – log (OR_DE_), where NIE = natural indirect effect (mediated pathway), TE = total effect, and DE = direct effect. The proportion of the total effect mediated by IPH was calculated using outcome-specific formulas. For the binary mortality outcome (a rare event), we applied the excess odds mediated formula: (OR_TE_-OR_DE_)/OR_TE_. For the ordinal mRS outcome, the proportion mediated was calculated on the log-odds scale as: (logOR_TE_ – logOR_DE_)/logOR_TE_. Confidence intervals for the proportion mediated were obtained by applying bias-corrected and accelerated bootstrap resampling with 5000 repetitions, stratified by the outcome to preserve its empirical distribution [44].

Given the high prevalence of venous infarction among patients with cytotoxic edema, we conducted additional Mediation analyses to evaluate its potential role in the association between cytotoxic edema and clinical outcomes.

All statistical analyses were deemed significant at a 2-sided α level of ≤ 0.05. We used the R statistical package (version 4.4.3; R Foundation for Statistical Computing, Vienna, Austria) for the analysis.

## Results

### Population Characteristics

A total of 394 hospitalized CVT patients (mean age 42.7 ± 16.9 years; 65.5% female) were included in the study. Demographics and baseline characteristics of both groups stratified by the presence of CE are summarized in Table 1.

**Table 1:** Baseline and treatment characteristics of the study sample

Patient Characteristics	Total (*N* = 394)	Cytotoxic Edema	
		+ *N* = 128 (32.5%)	- *N* = 266 (67.5%)
Admission Center, *N* (%)			
UIHC	273 (69.3)	92 (71.9)	181 (68)
INNNMVS	121 (30.7)	36 (28.1)	85 (32)
Age, in years			
Median (IQR)	40 [28–55]	42 [32.8–55.3]	39 [27.3 - 55]
Patient Sex, N (%)			
Female	258 (65.5)	84 (65.6)	174 (65.4)
Male	136 (34.5)	44 (34.4)	92 (34.6)
Race/ Ethnicity, N (%) *			
White	248 (63.3)	84 (65.6)	164 (62.1)
Black	13 (3.3)	3 (2.3)	10 (3.8)
Hispanic	125 (31.9)	37 (28.9)	88 (32.3)
Asian	6 (1.5)	4 (3.1)	2 (0.8)
Ordinal Baseline mRS, N (%) *			
0	343 (87.1)	113 (88.3)	230 (86.5)
1	29 (7.4)	8 (6.2)	21 (7.9)
2	8 (2)	2 (1.6)	6 (2.3)
3	7 (1.8)	1 (0.8)	6 (2.3)
4	3 (0.8)	0 (0)	3 (1.1)
5	3 (0.8)	3 (2.3)	0 (0)
Dichotomized mRS at Baseline, N (%) *			
0–2	380 (96.5)	123 (32.4)	257 (67.6)
3–5	13 (3.3)	4 (30.8)	9 (69.2)
BMI, kg/m2*			
Median (IQR)	28 [24.2- 32.5]	27.3[24–33.1]	28.6 [24.4–32.5]
GCS, N (%)*			
Mild (GCS 13–15)	323 (84.1)	94 (75.8)	229 (88.1)
Moderate (GCS 9–12)	26 (6.8)	12 (9.7)	14 (5.4)
Severe (GCS < 9)	35 (9.1)	18 (14.5)	17 (6.5)
Symptoms and Signs, N (%)			
Headache	301 (76.4)	94 (73.4)	207 (77.8)
Seizure	103 (26.1)	46 (35.9)	57 (21.4)
Dysarthria	38 (9.6)	14 (10.9)	24 (9)
Cranial Nerve Deficit	135 (34.3)	51 (39.8)	84 (31.6)
AMS	119 (30.2)	61 (47.7)	58 (21.8)
Motor Deficits	128 (32.5)	65 (50.8)	63 (23.7)
Sensory Deficits	77 (19.5)	40 (31.2)	37 (13.9)
Abnormal Coordination	25 (6.3)	10 (7.8)	15 (5.6)
Babinski	34 (8.6)	19 (14.8)	15 (5.6)
Normal Exam	121 (30.7)	23 (18)	98 (36.8)
Risk factors, N (%)			
Infection	53 (13.5)	11 (8.6)	42 (15.8)
Dehydration	72 (18.3)	30 (23.4)	42 (15.8)
Smoking	103(26.1)	29 (22.7)	74 (27.8)
Alcohol	134 (34)	47 (36.7)	87 (32.7)
History of Malignancy	34 (8.6)	9 (7)	25 (9.4)
History of Migraines	45 (11.4)	11 (8.6)	34 (12.8)
History of Systemic Disorders	48 (12.2)	16 (12.5)	32 (12)
Oral Contraceptive Use^╫^	77 (19.5)	21 (16.4)	56 (21.1)
Trauma	27 (6.9)	6 (4.7)	21 (7.9)
Imaging, N (%)			
Venous Infarct*	106 (28.6)	83 (64.8)	23 (9.5)
IPH*	111 (30.2)	89 (69.5)	22 (9.2)
SAH*	46 (12.6)	25 (19.8)	21 (8.8)
Clot Location, N (%)			
Cortical vein thrombosis*	99 (27.1)	44 (35.2)	55 (22.9)
Dural sinus thrombosis*	345 (93.2)	115 (89.8)	230 (95)
Deep cerebral venous system thrombosis*	40 (10.8)	16 (12.6)	24 (9.9)
Initial Medication, N (%) *			
Aspirin	12 (3.2)	3 (2.5)	9 (3.5)
LMWH	139 (37.3)	40 (33.6)	99 (39)
UFH	220 (59)	75 (63)	145 (57.1)
Warfarin	2 (0.5)	1 (0.8)	1 (0.4)
Endovascular Treatment, N (%) §	21 (5.3)	11 (8.6)	10 (3.8)
Decompressive craniectomy, N (%) §	16 (4.2)	12 (9.4)	4 (1.6)

*UIHC* University of Iowa Health Care, *INNNMVS* Instituto Nacional de Neurologia y Neurocirugía Manuel Velasco Suárez, *BMI* body mass index, *GCS* Glasgow Coma Scale, *IPH* Intraparenchymal hemorrhage, *SAH* Subarachnoid hemorrhage, *LMWH* Low-molecular-weight-heparin, *UFH* Unfractionated heparin, *mRS* modified Rankin Scale, *AMS* altered mental status

*Includes missing data for Race (0.5%; *n* = 2), BMI (1.8%; *n* = 7), GCS (2.5%; *n* = 10); mRS at Baseline (0.3%; *n* = 1), Venous Infarct (5.8%; *n* = 23), IPH (6.6%; *n* = 26), SAH (7.6%; *n* = 30), Clot location: Cortical veins thrombosis (*N* = 29), Dural sinuses thrombosis (*N* = 24), and Deep cerebral venous thrombosis (*N* = 25) Initial Medication (5.3%; *n* = 21)

§During the Index hospitalization

At presentation, a total of 323 patients had a GCS between 13 and 15 (84.1%), and most patients had a baseline mRS score of 0–2 (96.5%; *N* = 380). The most common presenting symptom was headache (76.4%; *N* = 301), followed by cranial nerve defect (34.3%; *N* = 135), motor weakness (32.5%; *N* = 128), altered mental status (30.2%; *N* = 119), and seizure (26.1%; *N* = 103). All patients received anticoagulation therapy, with unfractionated heparin (UFH) being the most used agent (59%; *N* = 220). All included patients underwent neuroimaging within 24 h of admission. CT was performed in all patients, and MRI was used as the primary imaging modality when available. MRI was obtained in 347 patients, all of whom also had CT, while CT alone was used in 47 patients in whom MRI was not performed (of those, global cerebral edema was identified in 11 patients, vasogenic edema in 4 patients, and cytotoxic edema in 5 patients). All MRI studies included either GRE or SWI sequences.

A total of 128 patients (32.5%) presented CE. These patients were older (42 [32.8–55.3] vs. 39 [27.3-55] years) and had a higher prevalence of seizures (35.9% vs. 21.4%), altered mental status (47.7% vs. 21.8%), motor deficit (50.8% vs. 23.7%), and sensory deficit (31.2% vs. 13.9%). Endovascular therapy (EVT) was performed in 11 CE patients (8.6%), and 12 patients (9.4%) underwent decompressive craniectomy. As expected, neuroimaging revealed a markedly higher prevalence of venous infarcts among patients with cytotoxic edema compared with those without CE (64.8% vs. 9.5%). IPH was also more frequently observed in the CE group, affecting 89 patients (69.5% vs. 9.2%). The dural sinuses were the most common site of thrombosis (*N* = 115; 89.8%).

### Edema Subtypes

Univariable regression analysis assessing outcomes based on cerebral edema subtypes is presented in Supplementary Table 3. All edema subtypes were significantly associated with higher mRS at discharge. However, at 6-month follow-up, only CE remained significantly associated with poor functional outcome (mRS 3–6), *p* < 0.001. Furthermore, CE was strongly associated with in-hospital mortality (aOR: 2.94; 95% CI: 1.13–8.28; *p* = 0.031) and significantly associated with 6-month mortality (aOR: 2.52; 95% CI: 1.04–6.41; *p* = 0.044). (Supplementary Table 4).

**Fig. 2:**
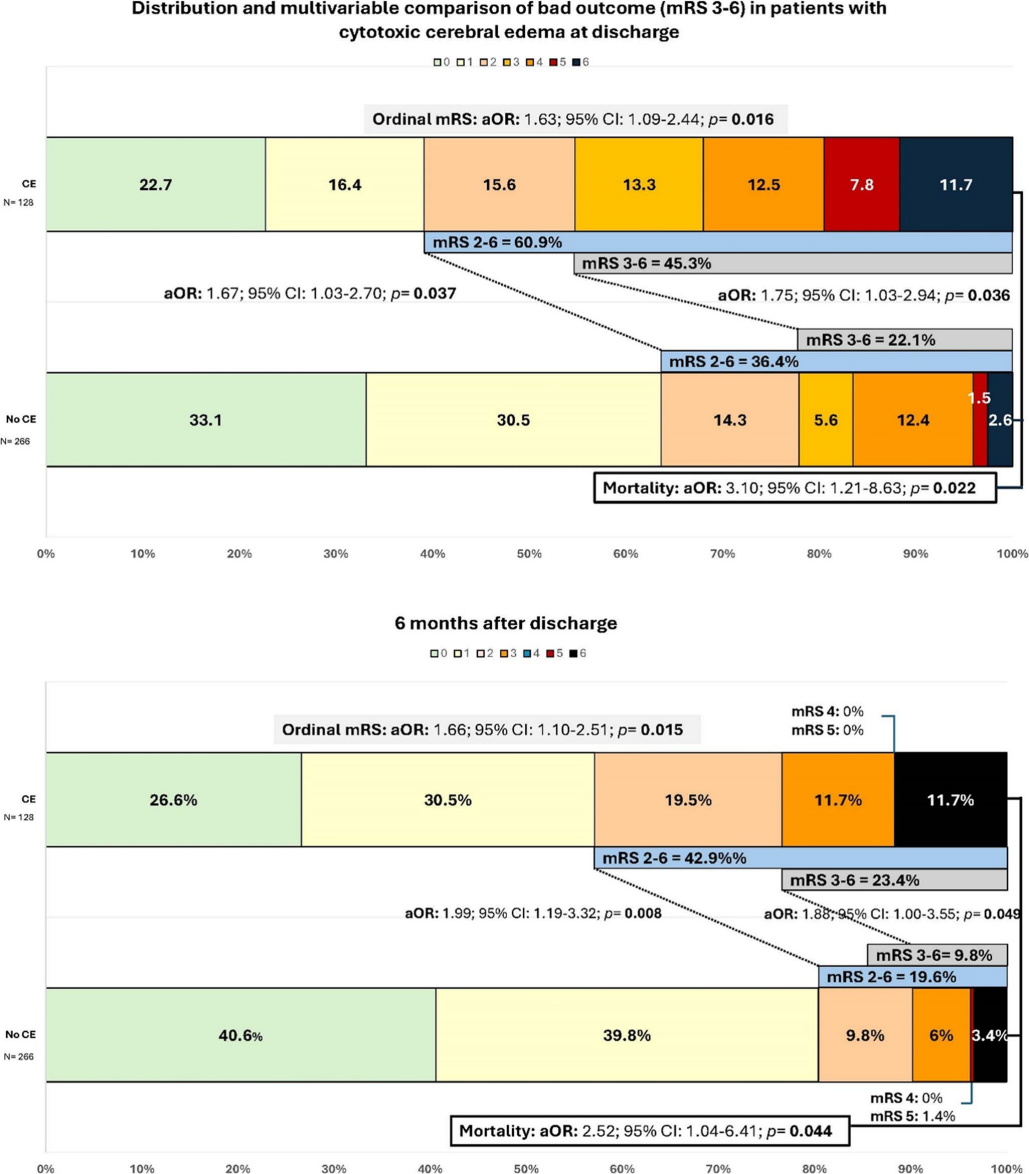
Distribution of mRS Scores at discharge and 6-month follow-up in patients with cytotoxic edema. CE: cytotoxic Edema, mRS: modified Rankin Scale, aOR: adjusted Odds Ratio. Legend. Ordinal mRS models: Adjusted for age, altered mental status, and motor weakness. Dichotomized mRS (0-2 vs 3-6): at 6 months after discharge, adjusted for age, motor weakness, and altered mental status, while at discharge, adjusted also for center. mRS 0-1 vs 2-6 at discharge: adjusted for motor weakness and altered mental status and initial anticoagulation, while at 6 months after discharge, adjusted also for age, altered mental status, and motor weakness. Mortality models: Adjusted for age and altered mental status

Figure 2 illustrates the mRS distribution among patients with CE, demonstrating a clear shift toward worse functional outcomes both at discharge and at 6 months. CE was significantly associated with poor functional outcome (mRS 3–6) at discharge and at 6 months (aOR: 1.75; 95% CI: 1.03–2.94; *p* = 0.036 & aOR: 1.88; 95% CI: 1.00–3.55; *p* = 0.049, respectively). In an additional analysis comparing less-than-excellent outcomes (mRS 2–6)) versus excellent (mRS 0–1, CE remained significantly associated with worse outcomes at both time points: discharge and at 6-month follow-up (aOR: 1.67; 95% CI: 1.03–2.70; *p* = 0.037 & aOR: 1.99; 95% CI: 1.19–3.32; *p* = 0.008, respectively).

### IPH as a Mediator of In-hospital Mortality

Mediation analysis demonstrated that CE was a significant predictor of in-hospital mortality (Pathway C, aOR 2.63; 95% CI, 1.01–7.12; *p* = 0.049), and was strongly associated with IPH (Pathway A, aOR 24.68; 95% CI, 13.43–47.63; *p* < 0.001), which in turn, independently predicted mortality even after adjusting for CE (Pathway B, aOR 8.73; 95% CI, 2.93–30.45; *p* < 0.001). However, when both CE and IPH were included in the same model, the direct effect of CE on mortality was no longer statistically significant (Pathway C′, aOR 0.61; 95% CI, 0.16–2.25; *p* = 0.458), suggesting that IPH mediated the relationship between CE and in-hospital mortality, with an estimated mediation effect of 76.8% of the total effect [42] (Table 2).

**Table 2:** Explained proportion and effect sizes in the mediation analysis. Outcome: In-Hospital Mortality Exposure: CE Mediator: Intra-parenchymal hemorrhage

In-Hospital Mortality				
Steps of analysis	Pathway	aOR	95% CI	*p*-value
I	C	2.63	1.01–7.12	**0.049**
II	A	24.68	13.43–47.63	**< 0.001**
III	B	8.73	2.93–30.45	**< 0.001**
IV	C’ (IPH) C’ (CE)	12.26 0.61	2.99–58.3 0.16–2.25	**0.001** 0.458
NDE NIE % of total effect mediated by IPH 95% CI for the proportion mediated			0.61 4.31 76.8% -1.702- 6.422	

*CE* Cytotoxic Edema, *IPH* Intraparenchymal hemorrhage, *aOR* adjusted odds ratio, *NDE* Net Direct Effect, *NIE* Net Indirect Effect

A multivariable regression analysis was adjusted for age, preexisting infection, center, GCS (Glasgow Coma Scale), and clot location

### IPH as a Mediator of Functional Outcomes at Discharge and 6-month Follow-up

Mediation analysis assessing the impact of CE on 6 months’ functional outcomes revealed a similar pattern. CE was significantly associated with higher mRS scores at follow-up (Pathway C, aOR 1.71; 95% CI, 1.06–2.74; *p* = 0.027), and remained strongly associated with IPH (Pathway A, aOR 24.85; 95% CI, 12.5-51.29; *p* < 0.001). IPH independently predicted worse functional outcome when controlling for CE (Pathway B, aOR 1.96; 95% CI, 1.18–3.25; *p* = 0.009). However, after adjusting for IPH, the direct effect of CE on mRS at 6 months became no longer significant (Pathway C′, aOR 1.09; 95% CI, 0.60–1.99; *p* = 0.777), suggesting that IPH also mediated this relationship, with an estimated mediation effect of 83.8% (Table 3).

**Table 3:** Explained proportion and effect sizes in the mediation analysis. Outcome: Ordinal mRS at 6 months Exposure: CE Mediator: Intra-parenchymal hemorrhage

Ordinal mRS at 6 months					
Steps of analysis	Pathway	aOR	95% CI		p-value
I	C	1.71	1.06 – 2.74		**0.027**
II	A	24.58	12.5 – 51.29		<**0.001**
III	B	1.96	1.18 – 3.25		**0.009**
IV	C’ (IPH) C’ (CE)	2.15 1.09	1.15 – 4.02 0.60 – 1.99		**0.016** 0.777
NDE NIE % of total effect mediated by IPH 95% CI for the proportion mediated				1.57 1.09 83.8% -0.859- 2.301	

*CE *Cytotoxic Edema, *IPH* Intraparenchymal hemorrhage, *aOR* adjusted Odds Ratio, *NDE* NetDirect Effect, NIE Net Indirect Effect

A multivariable regression analysis was adjusted for age, motor weakness, smoking, *NLR* Neutrophil Lymphocyte Ratio, *GCS* Glasgow Coma Scale, and Clot location

A similar mediation pattern was observed for functional outcome at discharge. While CE initially showed a significant association with higher mRS at discharge (Pathway C, aOR 1.77; 95% CI, 1.07–2.90; *p* = 0.025), this association lost significance after adjusting to IPH (Pathway C′, aOR 1.29; 95% CI, 0.69–2.40; *p* = 0.427), indicating that IPH also mediated the effect of CE on short-term functional outcome. (Supplementary Table 5).

Sensitivity analyses for the mediation analysis, excluding imputed data, yielded comparable results (Supplementary Tables 6 & 7). Results were also consistent in a separate sensitivity analysis excluding patients who did not undergo MRI (Supplementary Table 8).

Among patients with CE, venous infarction occurred in 83 cases and was not independently associated with the 6-month functional outcome. However, venous infarction demonstrated a mediating effect on mortality, which was smaller than that observed for intraparenchymal hemorrhage (Supplementary Table 9).

## Discussion

**Fig. 3:**
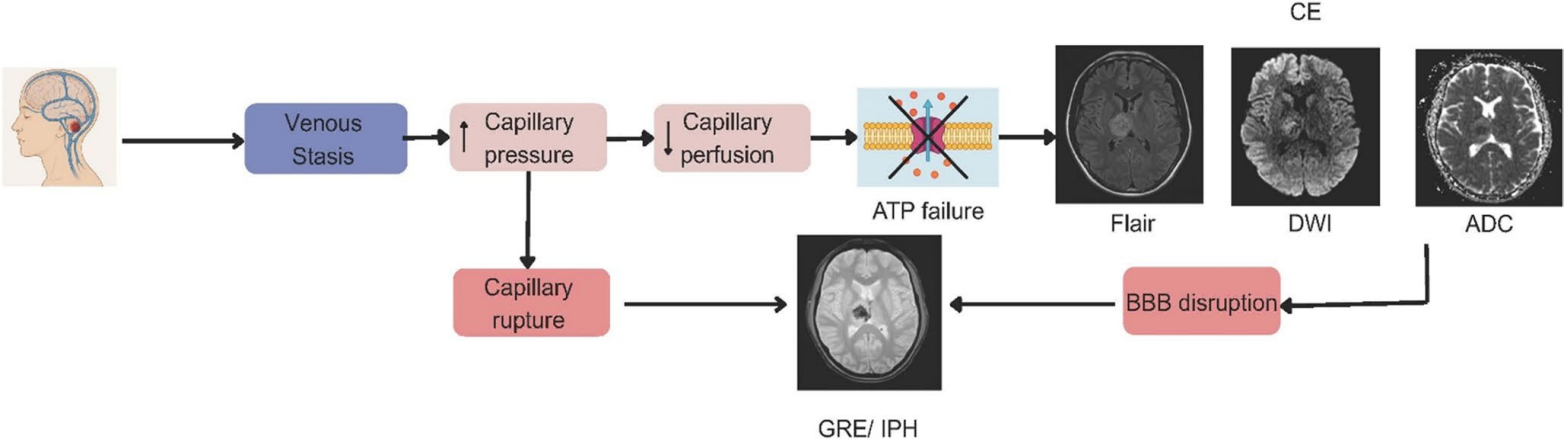
Pathophysiology of cytotoxic edema and intraparenchymal hemorrhage in cerebral venous thrombosis (CVT). CE= Cytotoxic Edema, BBB= Blood Brain Barrier, IPH= Intraparenchymal Hemorrhage. (Modified from Stam, 2005 & Forbes et.al., 2001) [13, 49]. Legend. This schematic illustrates the pathophysiological cascade and imaging correlates of CVT. Venous stasis leads to increased capillary pressure and reduced perfusion, resulting in ATP failure and subsequent CE, visible on MRI sequences as: Diffusion restriction from cytotoxic injury in DWI with corresponding hypointensity in ADC, confirming restricted diffusion. While the FLAIR sequence shows edema as hyperintensity. Elevated capillary pressure may also lead to capillary rupture, resulting in intraparenchymal hemorrhage (IPH), best visualized on GRE imaging. Also, BBB disruption as a result of CE further contributes to hemorrhage formation. * The diagram was generated by Nashwa Abdelhakim using Canva on May 30, 2025, for illustrative purposes. Patient imaging data used in this figure were obtained from participants enrolled in our study under IRB approval (202210065)

In our prior work, we demonstrated that among the edema subtypes seen in CVT: GCE, VE, and CE, only CE was significantly associated with in-hospital mortality and remained predictive of poor functional outcome at 3 and 6 months [10, 45]. Building on those findings, the current analysis suggests a theoretical pathway in which intraparenchymal hemorrhage likely mediates the effect of cytotoxic edema on both early mortality and functional disability in patients with cerebral venous thrombosis, but this could not be directly confirmed in this study. However, in previous experimental and clinical studies that support this temporal sequence, we suggest in this analysis that venous congestion results in CE, which, if unrecognized or untreated, may progress to IPH, a marker of irreversible brain injury and BBB disruption [11–13, 46–48]. This pathophysiological cascade, moving from venous congestion to CE, and subsequently to IPH, underscores the progressive nature of CVT (Fig. 3). Animal models consistently demonstrate this pattern. In a rat CVT model using the superior sagittal sinus (SSS) occlusion, it was shown that CE appears early, preceding hemorrhagic conversion, with histology confirming endothelial damage and extravasation [48]. Similarly, Li et al., in a combined SSS and cortical vein occlusion model, reported that all animals developed brain edema, and 59% progressed to hemorrhagic infarction [19]. These findings build upon earlier work by Forbes et al., who used DWI-MRI in 14 CVT patients and demonstrated that restricted diffusion, indicative of CE, is frequently observed in acute venous infarction and tends to resolve with time [13].

Previous clinical registry data support our findings. Simaan et al. reported increased incidence of IPH among CVT patients with venous infarction, isolated cortical vein thrombosis, and SSS involvement [50]. Pongmoragot et al. highlight that IPH complicates approximately one-third of CVT patients and is associated with poor functional outcomes [51]. They proposed a pathophysiological sequence where two mechanisms may occur simultaneously: thrombosis of the cerebral veins, causing infarction and petechial hemorrhage, and thrombosis of the major sinuses contributing to intracranial hypertension [51]. This aligns with our observations, as 75.9% of patients with venous infarction demonstrated hemorrhagic transformation.

In the International Study on Cerebral Vein and Dural Sinus Thrombosis (ISCVT) study published in 2004, a case fatality rate of 8% was reported, with IPH identified as one of the key risk factors for adverse outcomes [8]. Subsequent predictive tools, such as the ISCVT risk score (ISCVT-RS) [52] and the CVT Grading Scale (CVT-GS), incorporated IPH as a major component for stratifying patients [26]. In our study, after adjusting for IPH, the direct effect of CE on outcomes was no longer significant, strongly suggesting that the adverse effects of CE are largely mediated through early hemorrhagic transformation in the acute phase of CVT.

CE may represent an early and potentially reversible stage, but once it progresses to IPH, outcomes worsen substantially, consistent with severe endothelial disruption and irreversible tissue damage [12]. Importantly, VE, though frequent in CVT, appears to be a downstream phenomenon, arising after CE, as suggested by preclinical and clinical data [11, 12, 53]. This raises the possibility that early recognition of CE, given its potential reversibility, before venous infarction and hemorrhagic conversion, could be important in influencing the disease course, and highlights the need for further studies to clarify whether targeted interventions at this stage can improve outcomes. Notably, about one-third of poor outcomes in our cohort were not explained by IPH, suggesting other factors, such as the direct effect of CE, infarct location, collateral status, and other unmeasured confounders, may also contribute [54]. Future research should investigate these mechanisms.

### Limitations

Our study has several limitations. The study’s retrospective, observational design, and reliance on single-timepoint imaging limited the accuracy of symptom-onset to imaging intervals, preventing onset-based staging and introducing potential confounding from heterogeneous disease duration. Although our findings suggest a biologically theoretical pathway linking cytotoxic edema, intraparenchymal hemorrhage, and poor outcomes, the precise temporal relationship among these processes can not be definitively established within an observational framework. Because pre-admission imaging was unavailable, we could not confirm that CE preceded IPH; therefore, the mediation analysis should be interpreted as hypothesis-generating and consistent with, rather than confirmatory of, a sequential progression from cytotoxic injury to hemorrhagic transformation (Fig. 3). Future studies exploring this relationship should ensure imaging as early as symptoms start to better establish whether CE precedes IPH.

Additionally, 47 patients did not undergo MRI and were therefore assessed using CT. CE was identified in 5 of these patients, which may have introduced some heterogeneity. Reassuringly, a sensitivity analysis excluding patients without MRI data produced consistent findings. Also, exclusions from the analytic cohort were primarily driven by imaging availability rather than clinical characteristics or outcomes (e.g., patients with severe illness or early death may not have undergone imaging). As a result, the risk of selection bias cannot be fully excluded.

Outcome and covariate measurement limitations should be considered. The mRS was site-reported and therefore susceptible to interrater variability; however, neuroimaging was contemporaneously acquired and independently reviewed by blinded vascular neurologists, supporting the validity of radiological assessments. The use of pre-existing data may have led to incomplete or inconsistent documentation, which could have affected the accuracy of several variables, such as NIHSS at admission. Despite its clinical relevance as an indicator of initial CVT severity, NIHSS could not be reliably incorporated into multivariable regression analyses due to its structural unavailability in a substantial proportion of the cohort.

In addition, mediation analysis relies on key assumptions, particularly the absence of unmeasured confounding among the exposure, mediator, and outcome, which cannot be fully ensured in an observational dataset despite adjustment with clinically relevant covariates.

Finally, while our cohort is one of the largest to date with detailed imaging data, generalizability is particularly limited for less severe, outpatient-managed cases, and resource-limited settings with restricted MRI access. Future prospective studies with serial imaging are warranted to confirm the temporal evolution of these findings and to further elucidate the mechanistic relationship between cytotoxic and hemorrhagic injury in CVT.

## Conclusion

Our findings suggest that IPH likely mediates the effect of CE on early mortality and disability CVT, though this theoretical relationship could not be established in this study. These findings position CE not merely as a radiographic marker of injury, but as a potentially modifiable factor in the acute phase of CVT. Clinical implications include the utility of imaging-based surveillance to detect early CE in high-risk patients and guide timely escalation of therapy before hemorrhagic transformation occurs. Prospective studies evaluating targeted interventions in patients with early CE, prior to the development of IPH, represent an important next step toward improving outcomes in this population.

## Supplementary Information

Below is the link to the electronic supplementary material.

Supplementary Material 1

## Data Availability

The data supporting our results are available upon reasonable request to the corresponding author.
