# Supplementary material for: Cytotoxic Edema and Intra-parenchymal Hemorrhage: A Mediated Pathway to Mortality and Functional Outcome in Cerebral Venous Sinus Thrombosis- A sub-analysis of the CLOT-VENUS Registry
Source: Transl Stroke Res. 2026 Apr 14;17(2):41. doi: 10.1007/s12975-026-01426-9 (PMC13076434; doi:10.1007/s12975-026-01426-9)
Supplement: Supplementary file 1 — Supplementary Material 1 [file 12975_2026_1426_MOESM1_ESM.docx]

**Cytotoxic Edema and Intra-parenchymal Hemorrhage: A Mediated Pathway to Mortality and Functional Outcome in Cerebral Venous Sinus Thrombosis- A sub-analysis of the CLOT-VENUS registry**

**Translational Stroke Research journal**

Nashwa Abdelhakim, MD^1^; Milagros Galecio-Castillo, MD^1^; Piyush Kalakoti, MD^1^; Leonardo Cruz-Criollo, MD^1^; Aaron Rodriguez-Calienes, MD^1^; Anderson Brito, MD^1^; Jorge Cespedes, MD^1^; Amir Shaban, MD^1^; Anish Venkatesan, BS^1^; Vanessa Cano Nigenda, MD, MSc^2^; Andres Alberto Mercado Pompa, MD^2^; Nicholas M. Mohr, MD, MS ^3,4,5^; Adrian Pereda-Castillo, MD^2^; Kevin Enríquez Peregrino MD^2^, Hector Valdez Ruvalcaba MD^2^, Brian J. Smith, PhD^6^; James C. Torner, PhD^4^; Miguel A. Barboza, MD, MSc^7^; Antonio Arauz, MD, PhD^2^; Santiago Ortega-Gutierrez, MD, MSc^1,8,9^

^1^Department of Neurology, University of Iowa Health Care, Iowa City, IA, USA.

^2^Instituto Nacional de Neurologia y Neurocirugía Manuel Velasco Suárez, México City, México.

^3^Departments of Emergency Medicine, University of Iowa Carver College of Medicine, Iowa City, IA, USA.

^4^Department of Epidemiology, University of Iowa, College of Public Health, Iowa City, IA, USA.

^5^Division of Critical Care, Department of Anesthesia, University of Iowa Carver College of Medicine, Iowa City, IA, USA.

^6^Department of Biostatistics, University of Iowa Health Care, Iowa City, IA, USA.

^7^Department of Neuroscience, Hospital Dr. Rafael A. Calderon Guardia, San Jose, Costa Rica.

^8^Department of Neurosurgery, University of Iowa Health Care, Iowa City, IA, USA.

^9^Department of Radiology, University of Iowa Health Care, Iowa City, IA, USA.

**Corresponding Author:**

Santiago Ortega-Gutierrez, MD, MSc

Clinical Professor of Neurology, Neurosurgery, and Radiology

Director of the Cerebrovascular and Neurointerventional Laboratory

Program Director of the Endovascular Surgical Neuroradiology Fellowship Program

Office: 319-353-6629

Fax: 319-384-7199

Email: [santy-ortega@uiowa.edu](mailto:santy-ortega@uiowa.edu)

| **Supplementary Table 1.** Description of the CLOT-VENUS registry |
| --- |
| ***Description*** |
| The CoLlabOraTion on Cerebral VEnoUs ThrombosiS registry (CLOT-VENUS) is an international, retrospective observational multicenter collaboration involving two comprehensive academic stroke centers in the United States and Mexico. Inclusion criteria for this cohort were patients > 18 years of age, who were hospitalized with diagnoses of acute non-iatrogenic or traumatic cerebral venous thrombosis (CVT), and acute neuro-imaging studies available between 2004 and 2024. Although the INNNMVS registry was established in 1990, early data collection was limited to basic demographic variables without systematic imaging. Beginning in 2004, the INNNMVS transitioned to electronic data collection and storage, enabling secure digital archiving and sharing of clinical data and neuroimaging. In parallel, the institutional electronic medical record (EMR) and PACS systems at the University of Iowa were implemented during the same period, and consecutive cases with complete acute imaging became reliably available for review. Cases diagnosed before this period were excluded because imaging and clinical data were not consistently available or retrievable. The registry is funded by NIH/NINDS R03 (R03NS120228), whose primary objective is to identify early imaging (within the first 24h of admission), clinical, and laboratory biomarkers associated with poor outcomes, thereby informing risk stratification in CVT.  Acute CVT was defined as symptom onset within 30 days prior to presentation, clinical presentation suspicious for CVT with confirmatory diagnosis with magnetic resonance imaging (MRI) ± magnetic resonance venography (MRV) or computed tomography venography (CTV). Non-hospitalized patients (N=17) were also excluded, because standardized acute neuroimaging, in-hospital clinical assessments, and longitudinal outcome data were not consistently available. As a result, reliable evaluation of imaging-defined complications and functional outcomes was not feasible in these cases. All patients received standard-of-care management based on the most updated American Heart Association/ American Stroke Association guidelines.^1^ |

| **Supplementary Table 2.** mRS evaluations and Imputation Analysis Description |
| --- |
| ***Description*** |
| Discharge modified Rankin Scale (mRS) was derived from the standardized physical and occupational therapy evaluations performed prior to discharge. These assessments include structured documentation of independence in mobility, transfers, basic ADLs, communication/cognition, and supervision/assistance needs, which map directly to mRS categories. Two trained abstractors, blinded to imaging classifications and follow-up outcomes, applied a pre-specified mapping algorithm to assign the mRS; discrepancies were resolved by consensus. This method has been previously used and validated by our group.^2^ Follow-up mRS post-discharge was assessed during outpatient visits by certified clinical providers.^3^  Due to heterogeneity in follow-up mRS assessments’ timepoints, the mRS statuses at 6 months were imputed from the observed visits. In our dataset, mRS statuses at the two analytic timepoints were imputed from the observed visits.^4^ In particular, linear interpolation was used to impute mRS at analytic timepoints when bracketed by visits at which mRS was measured^4,5^ (N=394 [discharge]; 3 months N=295 [3 months]; and 275 [3 to 6 months]). mRS measured at the last visit was used when occurring two or fewer weeks before an analytic timepoint or in the case of death (N=18 [3 months]: N=22 [3 to 6 months]). Otherwise, analytic mRS was imputed with linear mixed effects regression that modeled mRS trends over time with patient-specific random intercepts and slopes (N= 81 [3 months]; N= 97 [3 to 6 months]). |

**Supplementary Table 3.** Univariate analysis in Patients with CVT based on the Cerebral Edema Subtype

| **Outcome measures** | **Global Cerebral Edema** | | | **Vasogenic Edema** | | | **Cytotoxic Edema** | | |
| --- | --- | --- | --- | --- | --- | --- | --- | --- | --- |
|  | **+** | **-** | ***p*-value** | **+** | **-** | ***p*-value** | **+** | **-** | ***p*-value** |
|  | **101 (25.63%)** | **293 (74.37%)** |  | **98 (24.9%)** | **296 (75.1%)** |  | **128 (32.5%)** | **266 (67.5%)** |  |
| **Discharge Outcomes (**394/394) | | | | | | | | | |
| mRS score 0 | 10 (9.9) | 107 (36.5) | **<0.001** | 19 (19.4) | 98 (33.1) | **0.016** | 29 (22.7) | 88 (33.1) | **<0.001** |
| mRS score 1 | 29 (28.7) | 73 (24.9) |  | 25 (25.5) | 77 (26) |  | 21 (16.4) | 81 (30.5) |  |
| mRS score 2 | 16 (15.8) | 42 (14.3) |  | 18 (18.4) | 40 (13.5) |  | 20 (15.6) | 38 (14.3) |  |
| mRS score 3 | 16 (15.8) | 16 (5.5) |  | 15 (15.3) | 17 (5.7) |  | 17 (13.3) | 15 (5.6) |  |
| mRS score 4 | 19 (18.8) | 30 (10.2) |  | 12 (12.2) | 37 (12.5) |  | 16 (12.5) | 33 (12.4) |  |
| mRS score 5 | 4 (4.0) | 10 (3.4) |  | 5 (5.1) | 9 (3) |  | 10 (7.8) | 4 (1.5) |  |
| mRS score 6 | 7 (6.9) | 15 (5.1) |  | 4 (4.1) | 18 (6.1) |  | 15 (11.7) | 7 (2.6) |  |
| **Functionally non-independent:** mRS 3-6 (vs 0-2) | 46 (45.5) | 71 (24.2) | **<0.001** | 36 (36.7) | 81 (27.4) | 0.078 | 58 (45.3) | 59 (22.2) | **<0.001** |
| **Excellent outcome:**  mRS 0-1 (vs 2-6) | 39 (38.6) | 180 (61.4) | **< 0.001** | 62 (63.3) | 215 (72.6) | **0.014** | 50 (39.1) | 169 (63.5) | **< 0.001** |
| **At 6 months** (394/394) | | | | | | | | | |
| mRS score 0 | 25 (24.8) | 117 (39.9) | **0.036** | 29 (29.6) | 113 (38.2) | 0.360 | 34 (26.6) | 108 (40.6) | **<0.001** |
| mRS score 1 | 42 (41.6) | 103 (35.2) |  | 41 (41.8) | 104 (35.1) |  | 39 (30.5) | 106 (39.8) |  |
| mRS score 2 | 18 (17.8) | 33 (11.3) |  | 13 (13.3) | 38 (12.8) |  | 25 (19.5) | 26 (9.8) |  |
| mRS score 3 | 7 (6.9) | 24 (8.2) |  | 11 (11.2) | 20 (6.8) |  | 15 (11.7) | 16 (6) |  |
| mRS score 4 | 0 (0) | 0 (0) |  | 0 (0) | 0 (0) |  | 0 (0) | 0 (0) |  |
| mRS score 5 | 1 (1) | 0 (0) |  | 0 (0) | 1 (0.3) |  | 0 (0) | 1 (0.4) |  |
| mRS score 6 | 8 (7.9) | 16 (5.5) |  | 4 (4.1) | 20 (6.8) |  | 15 (11.7) | 9 (3.4) |  |
| **Functionally non-independent:**  mRS 3-6 (vs 0-2) | 16 (15.8) | 40 (13.7) | 0.587 | 15 (15.3) | 41 (13.9) | 0.721 | 30 (23.4) | 26 (9.8) | **<0.001** |
| **Excellent outcome:**  mRS 0-1 (vs 2-6) | 67 (66.3) | 220 (75.1) | 0.088 | 70 (71.4) | 217 (73.3) | 0.717 | 73 (57.0) | 214 (80.5) | **< 0.001** |
| mRS: Modified Rankin Scale; Values within parentheses indicate the percentages. | | | | | | | | | |

**Supplementary Table 4.** Mortality for each cerebral edema at discharge and 6-month follow-up.

| **Mortality at discharge** | | |
| --- | --- | --- |
| **Type of edema** | **aOR (95% CI)** | ***p*-value** |
| Global Cerebral Edema | 1.14 (0.38-3.19) ^a^ | 0.806 |
| Vasogenic Cerebral Edema | 0.48 (0.13-1.45) ^a^ | 0.229 |
| Cytotoxic Cerebral Edema | 2.94 (1.13-8.28) ^b^ | **0.031** |
| **Mortality at 6 months** | | |
| **Type of edema** | **aOR (95% CI)** | ***p*-value** |
| Global Cerebral Edema | 1.62 (0.57-4.42) ^c^ | 0.347 |
| Vasogenic Cerebral Edema | 0.59 (0.17-1.60) ^c^ | 0.342 |
| Cytotoxic Cerebral Edema | 2.52 (1.04- 6.41) ^b^ | **0.044** |
| aOR = adjusted Odds Ratio.  ^a^ Adjusted for center, age, abnormal mental examination, and motor weakness.  ^b^ Adjusted for center, age, and abnormal mental examination.  ^c^ Adjusted for center, age, sex, seizure, and abnormal mental examination. | | |

**Supplementary Table 5.** Explained Proportion and Effect Sizes in the Mediation Analysis

Outcome: Ordinal mRS at discharge

Exposure: CE

Mediator: Intra-parenchymal hemorrhage

| **Discharge Ordinal mRS** | | | | | |
| --- | --- | --- | --- | --- | --- |
| **Steps of analysis** | **Pathway** | **aOR** | **95% CI** | | ***p-value*** |
| I | C | 1.77 | 1.07-2.90 | | **0.025** |
| II | A | 25.07 | 12.76-52.25 | | **<0.001** |
| III | B | 1.78 | 1.05-3.03 | | **0.034** |
| IV | C’ (IPH)  C’ (CE) | 1.73  1.29 | 0.90-3.33  0.69-2.40 | | 0.101  0.427 |
| NDE  NIE  % of total effect mediated by IPH  95% CI for the proportion mediated | | | | 1.29  1.37  55.6%  -4.289- 4.149 | |
| CE= Cytotoxic Edema; IPH= Intraparenchymal hemorrhage; aOR = adjusted Odds Ratio, NDE = Net  Direct Effect, NIE = Net Indirect Effect.  A multivariable regression analysis was adjusted for age, motor weakness, smoking, NLR (Neutrophil Lymphocyte Ratio), GCS (Glasgow Coma Scale), and Clot location | | | | | |

**Supplementary Table 6.** Explained Proportion and Effect Sizes in the Mediation Analysis in the non-imputed cohort

Outcome: Ordinal mRS at 6 months

Exposure: CE

Mediator: Intra-parenchymal hemorrhage

| **Ordinal mRS at 6 months** | | | | | |
| --- | --- | --- | --- | --- | --- |
| **Steps of analysis** | **Pathway** | **aOR** | **95% CI** | | ***p-value*** |
| I | C | 2.05 | 1.14-3.69 | | **0.0 0.017** |
| II | A | 28.06 | 12.24-70.6 | | **<0.001** |
| III | B | 1.89 | 1.01-3.53 | | **0.046** |
| IV | C’ (IPH)  C’ (CE) | 1.51  1.60 | 0.70-3.25  0.76-3.38 | | 0.297  0.216 |
| NDE  NIE  % of total effect mediated by IPH  95% CI for the proportion mediated | | | | 1.60  1.28  34.29%  -0.859-2.301 | |
| CE= Cytotoxic Edema; IPH= Intraparenchymal hemorrhage; aOR = adjusted Odds Ratio, NDE = Net Direct Effect, NIE = Net Indirect Effect.  A multivariable regression analysis was adjusted for age, motor weakness, smoking, NLR (Neutrophil Lymphocyte Ratio), GCS (Glasgow Coma Scale), and clot location. | | | | | |

**Supplementary Table 7.** Explained Proportion and Effect Sizes in the Mediation Analysis in the non-imputed cohort

Outcome: In-hospital Mortality

Exposure: CE

Mediator: Intra-parenchymal hemorrhage

| **In-hospital Mortality** | | | | | |
| --- | --- | --- | --- | --- | --- |
| **Steps of analysis** | **Pathway** | **aOR** | **95% CI** | | ***p-value*** |
| I | C | 3.07 | 1.06-9.53 | | **0.0 0.042** |
| II | A | 24.04 | 11.78-52.5 | | **<0.001** |
| III | B | 13.11 | 3.7-59.49 | | **<0.001** |
| IV | C’ (IPH)  C’ (CE) | 17.75  0.62 | 3.71-106.27  0.14-2.61 | | **0.001**  0.516 |
| NDE  NIE  % of total effect mediated by IPH  95% CI for the proportion mediated | | | | 0.62  4.93  79.7%  0.329-6.936 | |
| CE= Cytotoxic Edema; IPH= Intraparenchymal hemorrhage; aOR = adjusted Odds Ratio, NDE = Net  Direct Effect, NIE = Net Indirect Effect.  A multivariable regression analysis was adjusted for age, infection, center, Glasgow Coma Scale (GCS), and clot location. | | | | | |

**Supplementary Table 8.** Explained Proportion and Effect Sizes in the Mediation Analysis Restricted to Patients With MRI (n= 347)

Outcome: Ordinal mRS at 6 months

Exposure: CE

Mediator: Intra-parenchymal hemorrhage

| **Ordinal mRS at 6 months** | | | | | |
| --- | --- | --- | --- | --- | --- |
| **Steps of analysis** | **Pathway** | **aOR** | **95% CI** | | **p-value** |
| I | C | 1.72 | 1.06-2.80 | | **0.030** |
| II | A | 28.94 | 14.55-61.66 | | **<0.001** |
| III | B | 2.29 | 1.35-3.87 | | **0.002** |
| IV | C’ (IPH)  C’ (CE) | 2.75  0.95 | 1.42-5.33  0.51-1.78 | | **0.003**  0.872 |
| NDE  NIE  % of total effect mediated by IPH  95% CI for the proportion mediated | | | | 0.95  1.81  109%  0.246-7.336 | |
| CE= Cytotoxic Edema; IPH= Intraparenchymal hemorrhage; aOR = adjusted Odds Ratio, NDE = Net Direct Effect, NIE = Net Indirect Effect.  A multivariable regression analysis was adjusted for age, motor weakness, smoking, NLR (Neutrophil Lymphocyte Ratio), GCS (Glasgow Coma Scale), and clot location. | | | | | |

**Supplementary Table 9.** Mediation Analysis for the effect of venous infarction

Outcome: In-hospital mortality

Exposure: CE

Mediator: Venous infarction

| **In-hospital Mortality** | | | | | |
| --- | --- | --- | --- | --- | --- |
| **Steps of analysis** | **Pathway** | **aOR** | **95% CI** | | **p-value** |
| I | C | 2.67 | 1.03- 7.22 | | **0. 0.046** |
| II | A | 17.71 | 9.87- 33.19 | | **<0.001** |
| III | B | 2.55 | 0.97- 6.74 | | **0.055** |
| IV | C’ (VI)  C’ (CE) | 1.69  1.96 | 0.51- 5.72  0.58- 6.58 | | 0.392  0.273 |
| NDE  NIE  % of total effect mediated by VI | | | | 1.96  1.36  26.5% | |
| CE= Cytotoxic Edema; VI= venous infarction; aOR = adjusted Odds Ratio, NDE = Net Direct Effect, NIE = Net Indirect Effect.  A multivariable regression analysis was adjusted for age, infection, center, GCS (Glasgow Coma Scale), and clot location. | | | | | |

[**Supplementary**](#spfg1) **Figure 1.** Flow chart of the study population

**
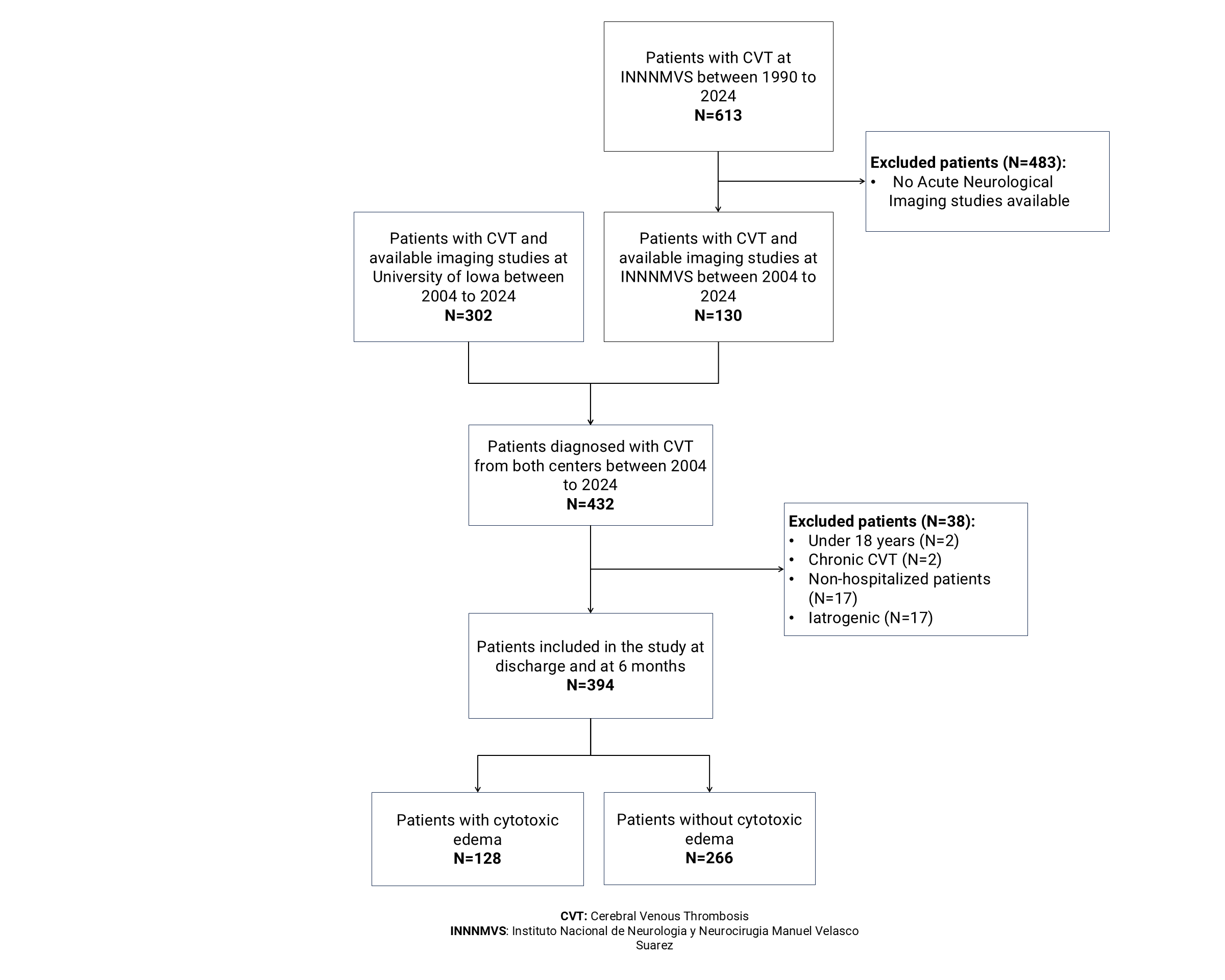
**

**References**

1. Saposnik G, Barinagarrementeria F, Brown RD, et al. Diagnosis and Management of Cerebral Venous Thrombosis. *Stroke*. 2011;42(4):1158-1192. doi:10.1161/STR.0b013e31820a8364

2. Ortega-Gutierrez S, Holcombe A, Aksan N, et al. Association of admission clinical predictors and functional outcome in patients with Cerebral Venous and Dural Sinus Thrombosis. *Clinical Neurology and Neurosurgery*. 2020;188:105563. doi:10.1016/j.clineuro.2019.105563

3. Bruno A, Akinwuntan AE, Lin C, et al. Simplified Modified Rankin Scale Questionnaire. *Stroke*. 2011;42(8):2276-2279. doi:10.1161/STROKEAHA.111.613273

4. Reeves KW, Stone RA, Modugno F, et al. A Method to Estimate Off-Schedule Observations in a Longitudinal Study. *Annals of Epidemiology*. 2011;21(4):297-303. doi:10.1016/j.annepidem.2010.11.013

5. Lepot M, Aubin JB, Clemens FHLR. Interpolation in Time Series: An Introductive Overview of Existing Methods, Their Performance Criteria and Uncertainty Assessment. *Water*. 2017;9(10):796. doi:10.3390/w9100796
